# Books

**Published:** 1883-06

**Authors:** 


### Books.

Transactions of Thirty-Third Annual Meeting of the American Medical Association. Held at St. Paul, Minn., June 6, 7, 8 and 9, 1882.

The following named gentlemen, appear as delegates from the Medical Association of Georgia: J. W. Bailey, Robert Battey, A. W. Calhoun, Henry F. Campbell, E. L.

Connally, J. D. Hamilton, W. F. Holt, Jas. Thad. Johnson, J. G. Thomas, J. S. Todd, W. F. Westmoreland.

Among the officers for 1883, the name of Dr. A. W· Calhoun appears as Chairman of the Section of Ophthalmology, Otology and Laryngology.

